# Association of Housing Disrepair Indicators with Cockroach and Rodent Infestations in a Cohort of Pregnant Latina Women and Their Children

**DOI:** 10.1289/ehp.7588

**Published:** 2005-07-27

**Authors:** Asa Bradman, Jonathan Chevrier, Ira Tager, Michael Lipsett, Jaqueline Sedgwick, Janet Macher, Ana B. Vargas, Elvia B. Cabrera, Jose M. Camacho, Rosana Weldon, Katherine Kogut, Nicholas P. Jewell, Brenda Eskenazi

**Affiliations:** 1 Center for Children’s Environmental Health Research, School of Public Health, University of California, Berkeley, California, USA; 2 School of Medicine, University of California, San Francisco, California, USA; 3 Clínica de Salud del Valle de Salinas, Salinas, California, USA; 4 Division of Environmental and Occupational Disease Control, California Department of Health Services, Oakland, California, USA; 5 Center for the Health Assessment of Mothers and Children of Salinas (CHAMACOS), Salinas, California, USA

**Keywords:** children, cockroaches, environment, exposure, Hispanic, home inspections, housing quality, Latino, pesticides, pregnant, rodents, women

## Abstract

Health burdens associated with poor housing and indoor pest infestations are likely to affect young children in particular, who spend most of their time indoors at home. We completed environmental assessments in 644 homes of pregnant Latina women and their children living in the Salinas Valley, California. High residential densities were common, with 39% of homes housing > 1.5 persons per room. Housing disrepair was also common: 58% of homes had peeling paint, 43% had mold, 25% had water damage, and 11% had rotting wood. Evidence of cockroaches and rodents was present in 60% and 32% of homes, respectively. Compared with representative national survey data from the U.S. Department of Housing and Urban Development, homes in our sample were more likely to have rodents, peeling paint, leaks under sinks, and much higher residential densities. The odds of rodent infestations in homes increased in the presence of peeling paint [odds ratio (OR) 2.1; 95% confidence interval (CI), 1.5–3.1], water damage (OR 1.9; 95% CI, 1.2–2.7), and mold (OR 1.5; 95% CI, 1.0–2.1). The odds of cockroach infestation increased in the presence of peeling paint (OR 3.8; 95% CI, 2.7–5.6), water damage (OR 1.9; 95% CI, 1.2–2.9), or high residential density (OR 2.1; 95% CI, 1.2–3.8). Homes that were less clean than average were more prone to both types of infestations. Pesticides were stored or used in 51% of households, partly to control roach and rodent infestations. These data indicate that adverse housing conditions are common in this community and increase the likelihood of pest infestations and home pesticide use. Interventions to improve housing and promote children’s health and safety in this population are needed.

The poor housing available to low-income families may be a chief contributor to persistent health disparities in the United States (Bashir 2002; Brugge et al. 2001; Crain et al. 2002; Kinney et al. 2002; Krieger and Higgins 2002; Krieger et al. 2002; Marsh 1982; Rauh et al. 2002; Thiele 2002). Deteriorated housing and its correlates can compromise many aspects of children’s health. For example, families in old or dilapidated homes suffer disproportionately from lead poisoning and from injuries due to household accidents (Bashir 2002; Marsh 1982; Shenassa et al. 2004). Structural deficiencies such as inadequate ventilation can contribute to dampness and mold growth, which cause or exacerbate respiratory morbidity (Bornehag et al. 2001, 2004; Brugge et al. 2001; Institute of Medicine Committee on Damp Indoor Spaces and Health 2004; Institute of Medicine Committee on the Assessment of Asthma and Indoor Air 2000; Peat et al. 1998; Ronmark et al. 1999; Spengler et al. 2004; Williamson et al. 1997). Poor housing conditions have been associated with infestations of rodents and cockroaches (Whyatt et al. 2002), both of which are allergenic, can carry infectious diseases (Baumholtz et al. 1997; Gubler et al. 2001), and can lead to increased use of home pesticides (Whyatt et al. 2002). The health burdens associated with poor housing may be particularly significant for young children, who spend the vast majority of their time inside their homes (California Air Resources Board 1991; Silvers et al. 1994).

To date, reports on housing quality have focused primarily on low-income homes in U.S. inner cities (Brugge et al. 2001; Crain et al. 2002; Kinney et al. 2002; Whyatt et al. 2002). Less attention has been paid to families in agricultural and rural communities. An unpublished report on farmworker housing prepared for the U.S. Department of Agriculture in 1980 identified severe housing shortages and substandard housing nationally (InterAmerica Research Associates, Inc. 1980). This report also documented a trend toward less employer-owned farmworker housing, leaving more farmworkers to compete for housing units on local rental markets. More recently, the Housing Assistance Council (HAC) coordinated a survey of 4,625 farm-worker homes nationwide (HAC 2001). Additionally, a community group conducted a questionnaire-based health and housing survey in the Salinas Valley and agricultural areas of Santa Cruz County, California (Applied Survey Research and the Center for Community Advocacy 2001). These studies document housing shortages, high rates of crowding, deteriorated conditions, and problems with affordability for low-income communities in agricultural areas.

In this study, we documented the housing quality in homes of Latino families with young children living in the Salinas Valley, an agricultural area in Monterey County, California. We investigated the association of housing disrepair indicators with cockroach and rodent infestations, evaluated the association of pest infestations and reported pesticide use, and examined the association of measured cockroach allergen levels and evidence of cockroach infestation to test the validity of our inspection methods.

## Methods

### Subjects and recruitment.

The Center for the Health Assessment of Mothers and Children of Salinas (CHAMACOS) is a longitudinal birth cohort study investigating environmental exposures and children’s health in the Salinas Valley, Monterey County, California, an agricultural community (Eskenazi et al. 2003). Study participants were recruited through Clinica de Salud del Valle Salinas and the Natividad Medical Center. These clinics serve a predominantly low-income, Latina clientele. Women entering prenatal care at these facilities between October 1999 and October 2000 were screened for eligibility. Women who were *a*) at ≤20 weeks gestation, *b*) qualified to receive poverty-based government health insurance, *c*) ≥18 years of age, and *d*) planning to deliver at the Natividad Medical Center, the local county hospital, were invited to participate in the study. Of 1,130 eligible women, 601 (53%) enrolled in the CHAMACOS study. Relative to women who declined enrollment, participants in the CHAMACOS study were more likely to be Mexican-born and Spanish-speaking and to have a household member working in agriculture.

Of the 601 study participants, 511 agreed to a home visit at enrollment (mean 13.4 ± 5.3 weeks gestation). Of these, 371 consented to a second home visit when their children reached approximately 6 months of age (mean 7.3 ± 1.3 months). Thirteen families did not complete an enrollment visit but were visited at 6 months postpartum. Seventy-seven participants did not complete a home visit at either enrollment or 6 months postpartum. Women who did not complete a home visit did not differ demographically from home visit participants. They were also no more or less likely than participants whose homes we visited to report pest infestation during the office-based baseline questionnaire.

For the 371 participants with two home visits, we selected homes for our sample according to the following criteria: If a participant moved between her baseline and 6-month home visit, each of her homes is present in our housing sample as a distinct home. If a participant did not move between visits, we used housing data from her prenatal visit. Our total sample consists of 644 homes inhabited by 524 distinct families. Written informed consent was obtained from all participants in accordance with procedures approved by the Committee for the Protection of Human Subjects at the University of California, Berkeley.

### Data collection.

Women were interviewed at enrollment in the study and again when their children were 6 months of age. All assessments were conducted in Spanish or English. Information collected included family demographics, household composition, and frequency of housecleaning. Shortly after each interview, study staff conducted a home inspection. The number of rooms in the household (excluding bathrooms, hallways, closets, or garages), the housing structure (i.e., detached home, duplex, multiunit apartment building), and the level of cleanliness were determined by direct observation. Cleanliness was scored on a three-point scale according to the amount of grease around stoves, the presence of dirty dishes and overflowing trash, and the presence of dust, dirt, and food particles on floors and behind cabinets, appliances, or furniture.

Indicators of home disrepair were determined by direct observation. The presence of mold was determined separately for the kitchen, living room, mother’s sleeping area, and child’s sleeping area. Mold was scored as “minimal” when growth was limited to crevices or small locations, “moderate” when growth covered < 1 m^2^ of wall space, and “extensive” when growth covered ≥1 m^2^ of wall space or was very thick in several areas. The most extensive mold growth in any room was used to represent the level of mold in the home. Water damage, leaks under sinks, peeling paint, rotting wood, and improperly vented appliances were coded as present or absent.

We measured wall moisture content in the child’s sleeping area and a central living area in 375 of the homes inspected 6 months after delivery, 130 of which are included in this analysis. Measurements were conducted with a pinless meter calibrated for sheetrock (Model CT100; Professional Equipment, Hauppauge, NY) at a point 45 cm above the floor at the horizontal midpoint of each wall. Additional measurements were conducted in areas that suggested water problems, such as heavy condensation on windows. Homes were coded as “damp” if the moisture level for any wall was ≥17%, the level at which the Monterey County Department of Health (Salinas, CA) recommends replacement of gypsum board.

Inspections for rodent infestations determined the presence or absence of mouse or rat feces, poison, or traps. Inspections for cockroaches determined the presence or absence of live or dead roaches or feces in typical habitats, including under sinks, along cabinet edges, and behind refrigerators. An infestation was considered active if we observed evidence of rodents or cockroaches or the participant reported their presence. We validated our roach infestation classification criteria by comparing cockroach allergen concentrations in a subset of homes with and without roaches.

We also completed a pesticide storage and use inventory for each home. Active ingredients were later confirmed with the California Product/Label Database, an Internet-based database operated by the California Department of Pesticide Registration (California Product/Label Database 2002).

### Dust sample collection and analysis.

Cockroach allergen concentrations [*Blatella germanica* (*Bla g1*)] were measured in house dust samples collected from a subset of 99 homes during the 6-month home visit. Two dust samples were collected from each home, one from the living area floor and one from the child’s bed or crib mattress. Samples were collected using a vacuum cleaner with a Medivac dust-sampling head (Medivac Healthcare Ltd., London, U.K.). The dust was collected on a 5-to 10-μg nylon screen after passing through a 0.3-mm prefilter. In the field, all samples were kept on ice with a desiccant. For allergen extraction, 50- to 100-mg aliquots of dust were centrifuged with a borate buffer solution (sodium chloride, boric acid, and sodium hydroxide dissolved in filtered deionized water). The samples were vortexed, mixed on an orbital rotator for ≥2 hr, and centrifuged for 25 min at 2,500 rpm and a temperature of 4°C. The supernatant was transferred to a vial and stored at –80°C until analysis. *Bla g1* allergens were measured by the IBT Reference Laboratory (Lenexa, KS) using monoclonal-based enzyme immunoassays with a detection limit of 0.60 U/g (Chapman et al. 1987, 1998; Pollart et al. 1991). We used the highest allergen concentration of the two samples to represent the levels in the home.

The 99 homes selected for allergen measurements comprise a nested case–control sample intended to compare the allergen levels in homes of children with and without lower respiratory symptoms. Because the homes in this subsample were not randomly selected, their allergen concentrations may not represent levels in the full CHAMACOS sample. For this article, we compared *Bla g1* allergen concentrations in homes with and without cockroach infestations to validate the inspection method.

### Data analysis.

We first computed the prevalence of adverse housing conditions. To investigate the association of housing disrepair indicators and pest infestations, we used contingency tables and odds ratios (ORs) to evaluate all two-by-two combinations of pest infestation and housing disrepair variables. Cockroach and rodent infestations were analyzed separately. For these analyses, the presence or absence of a pest infestation was used as the dependent variable. Indicators of housing disrepair were used as independent variables and included moderate or extensive mold or mildew, water damage, peeling paint, rotting wood, leaks under sinks, and, for the subset of homes with wall moisture measurements, moisture levels > 17%. Except for mold and mildew (coded as moderate or severe versus none or minimal), all housing disrepair indicators were coded as either present or absent.

We then developed multivariate logistic regression models using cockroach or rodent infestation as the dependent variables and housing disrepair variables and potential covariates (including building type, household characteristics, and demographic characteristics of the participant) as independent variables. Building type variables compared duplexes, multiunit apartments, and other residences (e.g., garages, trailers) to detached homes. Trailers were grouped separately from detached homes because trailer parks in the Salinas Valley are extremely dense and not comparable with detached homes with yards. Household characteristics included resident density (one or more persons per room versus less than one person per room), urbanicity (Salinas address versus living outside Salinas), household income relative to the year 2000 federal poverty level (scored as follows: poverty level or below = 1, less than 200% of poverty level = 2, 200% of poverty level or greater = 3), and level of cleanliness (more clean vs. less clean). Participants’ demographic characteristics included education level (never attended school = 1, grades 1–6 = 2, grades 7–9 = 3, grades 10–12 = 4, high school diploma or equivalent = 5, technical school = 6, some college = 7, college graduate or more = 8) and number of years in the United States (< 5 years vs. ≥5 years). We used backward-selection logistic regression to systematically evaluate and remove variables that did not significantly contribute to the overall model (*p* ≥0.10).

We also constructed a housing quality index according to methods used by Whyatt et al. (Whyatt et al. 2002). Each home was assigned an index value based on the total number of housing disrepair indicators present. Our index differed slightly from that of Whyatt and colleagues in that we did not include holes in ceilings or walls or recent loss of utility services, but did include rotting wood. Index values ranged from 0 to 5, with a score of 5 for homes with all five disrepair indicators present (peeling paint, water damage, moderate or extensive mold or mildew, rotting wood, and leaking sinks). We again developed multivariate logistic regression models using pest infestations as the dependent variable and the housing quality index score as the independent variable. To test the hypothesis that the log odds of pest infestation increased linearly with the number of housing disrepair indicators in a home, we used likelihood ratio tests to compare the models using continuous index scores with those containing individual indicator variables for each level (0–5) of disrepair. If the continuous model was not significantly different from the model with indicator variables (*p* ≥0.05), the linearity hypothesis was accepted.

To investigate whether pest infestations predicted home pesticide use, we constructed logistic regression models with infestation as the independent variable and home pesticide use as the dependent variable. We adjusted for the same covariates considered above.

Finally, to confirm the validity of our methods to identify cockroach infestations, we plotted the cumulative distributions of *Bla g1* allergen levels in homes with and without cockroaches and tested the equality of these distributions with the Kolmogorov-Smirnov test. We repeated these tests separately for the homes of children with and without asthma symptoms to ensure that the relationship between cockroach presence and allergen levels was independent of inhabitants’ respiratory health.

Our demographic description of the study population is based on data collected at the first interview. However, all analyses linking demographic characteristics to housing conditions or pest infestations use questionnaire data collected concurrently with the home visit in question. All analyses were conducted using Stata software, version 8.2 for Windows (StataCorp, College Station, TX).

## Results

### Demographic characteristics.

Table 1 summarizes demographic and household characteristics of the study population. Participants in this study were predominantly Mexican-born (85%), Spanish-speaking (93%) women living in poverty. The mean (± SD) age of participants was 26 ± 5 years of age at enrollment, and approximately half had resided in the United States for < 5 years at the time of enrollment. Most homes (88%) we visited were either detached homes or multiunit apartment buildings, and 69% of participants lived in a home with at least one agricultural worker. Although home ownership status was not assessed at the prenatal or 6-month visits, a subsequent survey revealed that nearly all CHAMACOS participants were renters. The demographic characteristics of the participants have been described in detail in previous papers (Eskenazi et al. 2003, 2004).

### Housing quality.

**Figure 1:**
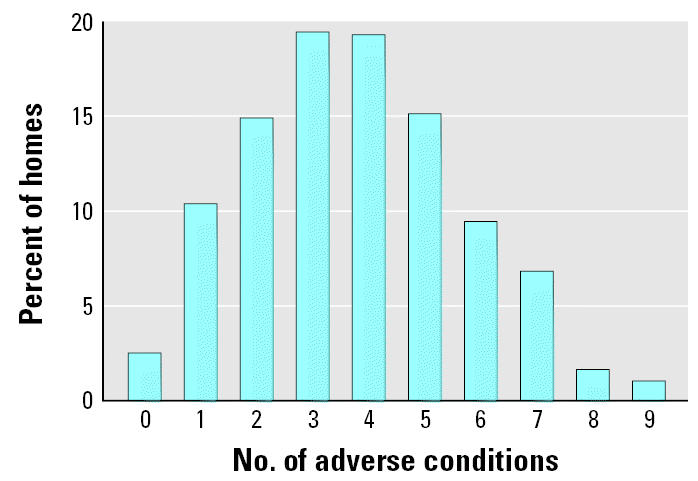
Percentage of homes with multiple adverse housing conditions within the CHAMACOS cohort.

Figure 1 and Table 2 summarize the housing quality characteristics of the 644 homes in this sample. Pest infestations were common, with 60% and 32% of homes containing cockroaches and rodents, respectively. Housing disrepair was also common; 58% of homes had peeling paint, 43% had mold, 25% had water damage, 16% had leaks under sinks, and 11% had rotting wood. Moderate or extensive mold was present in 28% of the sleeping areas used by participating children. High resident density was also very common, with 76% of participants living in homes with > 1 person per room and 39% with ≥1.5 persons/room. As shown in Figure 1, multiple adverse housing conditions were present in the majority of homes in this population, with < 3% of homes having no adverse conditions present.

### Housing characteristics and pest infestations.

Unadjusted ORs for each two-by-two combination of housing disrepair indicators, rodent infestation, and cockroach infestation are presented in Table 3. This univariate analysis is analogous to a correlation matrix, providing a measure of the association between the binary housing disrepair and pest infestations variables. Rodent infestation was strongly associated with cockroach infestation, peeling paint, water damage, rotting wood, and mold or mildew. Cockroach infestation was associated with every indicator of disrepair. Adverse housing conditions were strongly associated with each other.

Table 4 presents the final multivariate logistic regression models evaluating associations of housing disrepair with rodent and cockroach infestations. The presence of peeling paint [OR 2.1; 95% confidence interval (CI), 1.5–3.1], water damage (OR 1.9; 95% CI, 1.2–2.7), and moderate or extensive mold (OR 1.4; 95% CI, 1.0–2.1) were associated with increased odds of rodent infestations. Homes that were less clean than average were also associated with an increased odds of rodent infestations (OR 2.2; 95% CI, 1.0–4.7). Households in multiunit apartment buildings were less prone to rodent infestation than were detached homes (OR 0.6; 95% CI, 0.4–0.9).

The presence of peeling paint (OR 3.8; 95% CI, 2.7–5.6) and water damage (OR 1.9; 95% CI, 1.2–2.9) were also associated with increased odds of cockroach infestation. Other indicators of housing disrepair were not associated with cockroach infestation. Homes that were less clean than average had higher odds of cockroach infestation than cleaner homes (OR 3.7; 95% CI, 1.2–11.2). In contrast to the rodent infestation model, higher resident density was also associated with an increased odds of cockroach infestation (OR 2.1; 95% CI, 1.2–3.8). Homes in multiunit apartment buildings were more likely than detached homes to experience cockroach infestations (OR 3.0; 95% CI, 2.1–4.5). Households of recent immigrant women had higher odds of cockroach infestation than did households of women who had spent ≥5 years in the United States (OR 1.6; 95% CI, 1.1–2.4).

Each unit increase in the number of adverse housing conditions in a home was associated with an increased odds of both rodent and cockroach infestations (OR 1.5; 95% CI, 1.3–1.7 for rodents; OR 1.7; 95% CI, 1.5–2.0 for roaches). Based on evaluation of the maximum likelihood ratio, the log odds of rodent infestation increased with the total number of housing problems in a linear fashion (χ;^2^ = 3.9, df = 4, *p* = 0.4), whereas the log odds of cockroach infestation did not increase linearly (χ;^2^ = 16.0, df = 4, *p* = 0.003) (not shown).

### Home pesticides inventory.

Pesticides were stored in 313 (49%) of the 644 homes. Respondents in an additional 14 homes reported having used pesticides that were no longer present in the home; conversely, respondents in 18 homes with stored pesticides reported not having used them. Overall, 309 (48%) households reported home pesticide use. Of the 644 homes in our study, 31% stored pyrethroids, 9% stored piperonyl butoxide, 6% stored carbamates, 5% stored organophosphates, 4% stored hydramethylnon, and 4% stored boric acid. Spray-application pesticides were present in 30% of homes, pellets or powders in 10% of homes, and roach bait stations in 6% of homes. Pesticide gels, bombs, or rodent food imitators were present in < 5% of homes. In the 6 months preceding the visits, professional pesticide applications to control insects had been conducted in 5% of the homes; only two homes were reported to have been professionally treated for rodents in the same timeframe. As expected, insecticide use was more common in homes with cockroach infestations (OR = 2.4; 95% CI, 1.7–3.4) than in homes without such infestations (not shown).

### Cockroach allergen concentrations.

**Figure 2:**
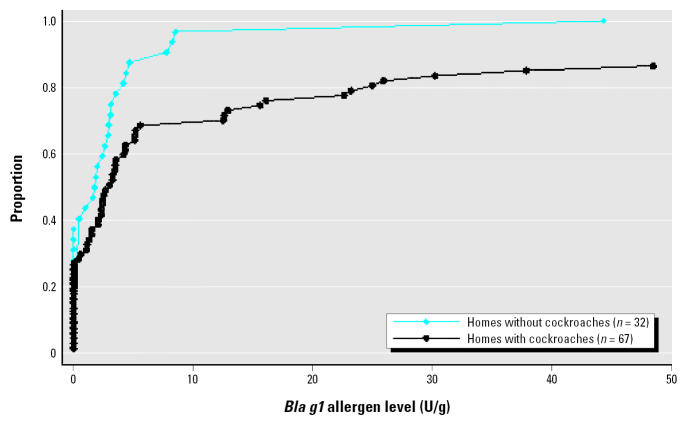
Cumulative distribution of cockroach allergen levels for CHAMACOS homes with and without identified cockroach infestation (excludes nine values > 50 U/g in cockroach-infested homes).

Figure 2 compares cockroach (*Bla g1*) allergen concentrations in a subset of 99 homes with and without identified cockroach infestations. Cockroach allergen concentrations were significantly higher in homes with evidence of infestations than in homes without infestation [median (interquartile range) = 3.0 (< 0.6–16.1) U/g for homes with cockroaches and 1.8 (< 0.6–3.4) U/g for homes without cockroaches; Kolmogorov-Smirnov statistic D = 0.28, *p* = 0.04], providing an external validation of our observations. The relationship between infestation and elevated cockroach allergen concentration was the same in the homes of children with and without asthma symptoms (data not shown).

## Discussion

This investigation is the first population-based cohort study documenting the housing conditions of low-income, Latino families in a U.S. agricultural community. Adverse housing conditions were common in this population. Pest infestations, mold and mildew, water damage, peeling paint, leaks, rotting wood, and high residential density were widespread, with multiple problems occurring in the vast majority of homes. Many of the conditions are markers of building dampness (e.g., water damage), sources of clinically important allergens (e.g., cockroach infestations), or respiratory irritants (e.g., volatile organic compounds generated by mold metabolism). As reviewed in the introduction, building dampness, allergens, and respiratory irritants have been associated with cough, wheeze, and increased asthma symptoms and may be etiologically related to the development of asthma in children. Rodents and cockroaches are also potential carriers of infectious diseases. The level of overcrowding in these homes may pose an additional threat to children’s health, as infectious diseases can spread rapidly among individuals who share close living quarters.

Our findings on housing quality characteristics are consistent with available data for low-income agricultural populations and some urban populations (Crain et al. 2002; Whyatt et al. 2002) (Table 2). Our ability to observe infestations during home inspections may explain the higher prevalence of cockroach and rodent infestations we reported compared with the questionnaire-based survey conducted by the Center for Community Advocacy (CCA) (Table 2) (Applied Survey Research and the Center for Community Advocacy 2001). It is also possible that our methods overestimated the prevalence of live cockroach infestations because the presence of dead roaches or feces, which we defined as evidence of a current infestation, may reflect past infestations that were no longer active. However, our finding that cockroach allergen levels were higher in homes with evidence of cockroaches adds validity to our findings. The higher frequency of leaks reported in the CCA survey is likely due to their inclusion of questions about leaking faucets, which we did not record. The high prevalence of mold infestations in this study may be related to the damp, cool winters in this region, poor building quality, and household crowding, which increases ambient moisture from respiration, cooking, and bathing. Compared with national data for Hispanic households, peeling paint, rodent infestations, and leaks under sinks were more common in our sample (Table 2). Especially striking in the CHAMA-COS population was the much higher resident density; 39% of homes had ≥1.5 people per room. By comparison, only 3% of Hispanic households and 0.5% of all U.S. households experience this level of crowding (U.S. Census Bureau 2002).

Pest infestations in the homes we inspected were consistently associated with housing disrepair indicators. Our findings are very similar to the 30–60% increase in the odds of pest infestation reported to be associated with each additional adverse housing condition in New York (Whyatt et al. 2002). However, the use of a simple, linear housing disrepair index may not be appropriate for statistical analyses relating housing conditions to pest infestations. In our data, the odds of cockroach infestation did not increase linearly with the number of adverse housing conditions. This nonlinearity underscores the need to assess the shape of the relationship between environmental index scores and epidemiologic outcomes, particularly when the scale is previously untested.

Peeling paint and water damage were each independently associated with pest infestations. Whereas water damage may indicate a source of water for pests, it is unlikely that peeling paint “causes” infestation. Rather, these conditions, both of which were associated with other housing disrepair indicators, may simply be indicators of building conditions that create favorable habitats for pests. The finding that cockroaches are more common in multiunit apartment buildings is consistent with other studies (Chew et al. 1998; Kitch et al. 2000; Leaderer et al. 2002) and is not surprising, given that each infested apartment in the building is a potential source of infestation for adjacent households. The finding that rodent infestations are less common in multiunit apartments than in detached homes may be due to the number of stories between the housing unit and ground level. Although most detached homes in our study are single story, potentially offering multiple routes of ingress to ground-dwelling rodents, the apartment buildings we visited are generally one to three stories. It is possible that the distance from ground level offers protection to residents of second- and third-story units. Our finding that less-clean households are more prone to pest infestation reflects the fact that cleaner homes offer pests fewer sources of food and water (e.g., crumbs and spills on the floor). Although the vast majority of study participants frequently clean their homes, their ability to maintain better housekeeping was compromised by poor building conditions and crowded households.

About half of the families we visited used pesticides to control pests in their homes. Insecticides were used in much greater quantities than rodenticides. The high proportion of pyrethroid insecticides likely reflects industry efforts to substitute pyrethroids for organophosphate pesticides, which were recently banned for home use by the U.S. Environmental Protection Agency (EPA) (U.S. EPA 2000a, 2001). The CHAMACOS families most commonly used insecticide sprays and powders, which have a higher exposure risk compared with bait stations and gels. Hydramethylnon roach gels, which can be strategically placed out of children’s reach, were used in only a small minority of households.

There are several limitations to the analyses presented here. Study participants differed somewhat from families that declined enrollment. Thus, our findings may not be generalizable to all low-income families residing in the Salinas Valley. However, the consistency of our findings with a previous questionnaire-based survey (Applied Survey Research and the Center for Community Advocacy 2001) suggests that the housing problems we have identified represent typical conditions for low-income families in this community. Another limitation is that the associations we found between housing disrepair and pest infestations do not necessarily reflect causal relationships. As noted above, housing disrepair indicators may be proxies for the overall condition of the building and not specific building characteristics that cause pest infestations. Additionally, the population was uniformly low income. Pest infestations may be related to multiple social and physical factors that could confound the association between housing characteristics and pest infestations. For example, crowded, low-income neighborhoods in our study area may receive fewer public services such as neighborhood pest control and housing code enforcement.

Access to adequate housing is considered a basic human right (United Nations 2005). Our findings indicate that housing is inadequate in this population. Interventions to improve housing quality should focus both on individual-level behaviors and policies to improve access to quality housing. Successful interventions to reduce cockroach infestations have used integrated pest management techniques (Brenner et al. 2003). Additional research is needed to identify the best combination of physical interventions, least-toxic pest control measures, and educational strategies that are sustainable in this population. These interventions will need to be low- or no-cost and accessible to a Spanish-speaking population. Given the high use of pesticide sprays and powders in our population, a first step could be the promotion of baited roach gels, which effectively control roach populations but are less likely to expose children (Brenner et al. 2003; Schechner 2004). Other successful strategies include programs to strengthen renters’ ability to negotiate housing improvements with landlords (Krieger and Higgins 2002).

We recognize that many factors, including overcrowding and deteriorated building conditions, are beyond the control of individual, low-income families. At the county and state levels, land use and housing policies should support construction of high-quality, affordable housing. Additionally, programs to improve housing conditions should be strengthened, including increased inspections.

Our findings have several implications for national housing policy. Although young children spend most of their time inside their homes, housing quality is not currently included in the children’s environmental quality indicators tracked by the U.S. EPA (U.S. EPA 2000b). National housing quality data are currently compiled by the U.S. Department of Housing and Urban Development (HUD) (U.S. Census Bureau 2003) and could be incorporated into the U.S. EPA tracking program. Recently, Healthy People 2010 has established specific goals related to housing quality, including reducing indoor allergen levels and decreasing the proportion of families that live in substandard housing (U.S. Department of Health and Human Services 2000). The HUD Healthy Homes Initiative, created in 1997, is developing programs to support these goals. We suggest that progress on the Healthy People 2010 housing quality objectives should be monitored by distinct regions and populations to ensure that the housing quality of vulnerable groups, such as those living in low-income agricultural regions, are not averaged into larger populations with fewer problems. Given that an overarching goal of U.S. federal health and environmental agencies is to reduce health disparities (U.S. Department of Health and Human Services 2000), efforts to improve housing should be prioritized as a children’s environmental health concern with substantial opportunities for success.

## Figures and Tables

**Table 1:** Demographic characteristics of CHAMACOS families who participated in home visits at enrollment or 6 months postpartum (*n* = 524).^a^

Characteristic	No. (%)
Mother’s country of birth	
Mexico	445 (84.9)
United States	65 (12.4)
Other	11 (2.1)
Not reported	3 (0.6)
Years mother has resided in United States^b^	
< 5	250 (47.7)
≥5	274 (52.3)
Mother’s highest level of education	
Some elementary school (grades 1–6) or less	226 (43.1)
Some secondary school (grades 7–12)	191 (36.5)
High school graduate or equivalent	59 (11.3)
Some education beyond high school	48 (9.2)
Language spoken at home	
Spanish	462 (88.2)
Spanish and English	24 (4.6)
English	29 (5.5)
Not reported	9 (1.7)
Family income relative to federal poverty level^c^	
≤Poverty level	302 (57.6)
> Poverty level but < 200% poverty level	170 (32.4)
≥200% poverty level	21 (4.0)
Not reported	31 (5.9)
Housing type^d^	
Detached home	275 (42.7)
Duplex (two apartments)	33 (5.1)
Multiunit apartment building (three or more apartments)	290 (45.0)
Other (e.g., garage, trailer)	45 (7.0)
Not reported	1 (0.2)
No. of household members	
1–3	73 (13.9)
4–6	220 (42.0)
≥7	225 (42.9)
Not reported	6 (1.2)
Agricultural workers in home	
0	140 (26.7)
1–3	273 (52.1)
≥4	88 (16.8)
Not reported	23 (4.4)
Frequency of housecleaning^e^	
Daily	443 (84.5)
Several times per week	73 (13.9)
Once per week to once every 2 weeks	6 (1.2)
Not reported	2 (0.4)
Level of cleanliness in home	
More clean	492 (93.9)
Less clean	30 (5.8)
Not rated	2 (0.4)

a Demographic characteristics reported for individual families that permitted home visits either at enrollment or 6 months postpartum (*n* = 524). These distributions are nearly identical to household characteristics for the total sample of 644 distinct homes that includes 131 movers (see text).

b Mother’s years in United States at time of entry into CHAMACOS project.

c Families’ poverty levels were calculated using the U.S. Department of Health and Human Services thresholds for the year 2000. A family of four with an annual income of ≤$17,050 was considered to be at or below the poverty level; the same family earning between $17,051 and $34,100 is within 200% of the poverty level.

d Building type for 644 distinct homes that were inspected.

e Defined as frequency with which the floor most often cleaned is mopped or vacuumed.

**Table 2:** Adverse housing conditions (%) in the CHAMACOS cohort and other populations.

Home characteristic	CHAMACOS (*n* = 644)	Local Farmworker Survey^a^ (*n* = 780)	NYC Cohort^b^ (*n* = 316)	HAC Survey^c^ (*n* = 4,625)	Hispanic U.S.^d^ (*n* = 9,814)	All U.S.^d^ (*n* = 106,261)
Rodents	32	18	53	19^c^	11	8
Cockroaches	60	48	66	19^c^	—	—
Pesticides stored in home	49	—	85	—	—	—
Peeling paint	58	33	42	29	4^e^	3^e^
Leak under sink	16	34^a^	22	—	5^f^	4^f^
Gas stove without functional vent^g^	35	—	—	—	—	—
Water damage	25	—	21	29	—	—
Rotting wood	11	—	—	—	—	—
Moderate or extensive mold anywhere in home	43	—	17	—	—	—
Moderate or extensive mold in child’s sleeping area^h^	28	—	—	—	—	—
Wall moisture > 17%^i^	26	—	—	—	—	—
Density (persons/room)						
≤0.5	2	—	—	—	42	70
0.51–1.00	22	—	—	—	45	28
1.01–1.50	37	—	—	74.2 (> 1.0)^j^	10	2
≥1.51	39	—	—	—j	3	0.5

—, data not available.

a Data from Applied Survey Research (2001): questionnaire-based; data for leaks include faucets.

b Data from Whyatt et al. (2002): questionnaire-based; pregnant African-American and Dominican women.

c Data from HAC (2001): 19% is the proportion of homes with unsanitary conditions, including rodent and insects.

d Department of Housing and Urban Development (HUD 2001) survey of occupied U.S. homes: questionnaire-based.

e HUD data are for peeling paint and broken plaster.

f HUD data are for plumbing leaks anywhere in house.

g Includes gas stoves without vents and or with nonfunctioning vents.

h Only applicable at 6-month visit (*n* = 133);

i Measured in 130 homes at 6-month visit; the Monterey County Health Department suggests sheetrock replacement if moisture > 17%.

j Proportion of units with children where density exceeded 1 person per room.

**Table 3:** OR matrix showing the interrelationships of housing disrepair indicators and pest infestations^a^ (*n* = 619–644^b^).

	Rodents	Cockroaches	Peeling paint	Water damage	Rotting wood	Mold	Leak under sink
Cockroaches	3.4**						
Peeling paint	2.4**	4.2**					
Water damage	2.5**	2.2**	2.1**				
Rotting wood	2.2**	2.2**	6.0**	8.4**			
Mold	2.0**	1.7**	1.9**	6.4**	4.3**		
Leak under sink	1.4	2.1**	2.2**	4.0**	7.5**	2.2**	
High density	1.1	2.7**	2.1**	2.5*	1.2	1.9*	1.1

a All variables are binary, with high density defined as > 1 person per room. ORs provide a measure of the association between the variables. We used this measure in lieu of Pearson or Spearman correlation coefficients, which are not applicable to binary variables.

b Number ranges from 619 to 644 depending on the number of missing values.

* *p* < 0.05.

** *p* < 0.01.

**Table 4:** Association of housing disrepair indicators with rodent and cockroach infestations: results of logistic regression models [OR (95% CI)].^a,b^

Home characteristic	Rodent infestation (*n* = 640)	Roach infestation (*n* = 629)
Peeling paint		
No	1.0	1.0
Yes	2.1 (1.5–3.1)	3.8 (2.7–5.6)
Water damage		
No	1.0	1.0
Yes	1.9 (1.2–2.7)	1.9 (1.2–2.9)
Mold		
None or minimal	1.0	—c
Moderate or extensive	1.5 (1.0–2.1)	
Resident density		
< 1 person/room	—c	1.0
≥ 1 person/room		2.1 (1.2–3.8)
Housing type		
Detached home	1.0	1.0
Duplex	0.9 (0.4–2.0)	0.9 (0.4–2.0)
Multiunit building^d^	0.6 (0.4–0.9)	3.0 (2.1–4.5)
Other^e^	0.9 (0.4–1.8)	0.9 (0.4–1.8)
Level of cleanliness in home		
More clean	1.0	1.0
Less clean	2.2 (1.0–4.7)	3.7 (1.2–11.2)
Years in United States		
≥5	—c	1.0
< 5		1.6 (1.1–2.4)

a See “Methods” for definition of rodent or cockroach infestation.

b Covariates considered as confounders and found insignificant for rodent and cockroach infestations included maternal education level, household income, and urbanicity.

c Resident density and years in United States were not associated with rodent infestation, and mold was not associated with cockroach infestations; these variables were not included in final models for these infestations.

d Apartment building with ≥3 units.

e Includes mobile homes, converted garages, a camp in the fields, and a home inside a business. Does not include detached homes, which serve as the reference group. See “Methods” for justification.
